# Effect on short‐term outcomes of the COVID‐19 pandemic following laparoscopic distal gastrectomy and low anterior resection for gastric and rectal cancer: A retrospective study using the Japanese National Clinical Database, 2018–2022

**DOI:** 10.1002/ags3.12901

**Published:** 2024-12-23

**Authors:** Masafumi Inomata, Hideki Endo, Tomonori Akagi, Hidefumi Shiroshita, Shigeki Yamaguchi, Susumu Eguchi, Norihito Wada, Yukinori Kurokawa, Yosuke Seki, Yoshiharu Sakai, Hiroyuki Yamamoto, Yoshihiro Kakeji, Yuko Kitagawa, Akinobu Taketomi, Masaki Mori

**Affiliations:** ^1^ Academic Committee of Japan Society for Endoscopic Surgery Tokyo Japan; ^2^ Department of Gastroenterological and Pediatric Surgery Oita University Faculty of Medicine Oita Japan; ^3^ Department of Healthcare Quality Assessment, Graduate School of Medicine The University of Tokyo Tokyo Japan; ^4^ Department of Surgery, Division of Colorectal Surgery Tokyo Women's Medical University Tokyo Japan; ^5^ Department of Surgery Nagasaki University Graduate School of Biomedical Sciences Nagasaki Japan; ^6^ Department of Medical Engineering Kobe University Graduate School of Medicine Kobe Japan; ^7^ Department of Gastroenterological Surgery Osaka University Graduate School of Medicine Osaka Japan; ^8^ Weight Loss and Metabolic Surgery Center Yotsuya Medical Cube Tokyo Japan; ^9^ Japanese Red Cross Osaka Hospital Osaka Japan; ^10^ Division of Gastrointestinal Surgery, Department of Surgery Kobe University Graduate School of Medicine Kobe Japan; ^11^ Department of Surgery Keio University School of Medicine Tokyo Japan; ^12^ Department of Gastroenterological Surgery I Hokkaido University Graduate School of Medicine Sapporo Japan; ^13^ The Japan Surgical Society Tokyo Japan

**Keywords:** COVID‐19, laparoscopic surgery, National Clinical Database

## Abstract

**Aims:**

We previously reported no change in surgical outcomes for laparoscopic distal gastrectomy (LDG) and laparoscopic low anterior resection (LLAR) early in the COVID‐19 pandemic (2020), although the number of elective surgeries decreased. In 2021, COVID‐19 spread further, with vaccination and other medical measures based on several medical societies' guidelines being initiated. Using the Japanese National Clinical Database (NCD), we added 2022 data to the 2018–2021 data to analyze the impacts of expansion of the COVID‐19 infection and its spread on laparoscopic surgery (including robot‐assisted surgery).

**Methods:**

Data on patients who underwent LDG and LLAR for cancer were extracted from the NCD between 2018 and 2022. The numbers of LDG and LLAR were obtained, and morbidity and mortality rates were evaluated using a standardized morbidity/mortality ratio (SMR), i.e. the ratio of the observed number of incidences to expected number of incidences calculated by the risk calculator previously developed by the NCD.

**Results:**

The numbers of LDG and LLAR cases declined in 2020, the first pandemic year, and continued to decline in 2022 to the same level as 2021, but with no further decline and no recovery trend in the number of cases. Numbers of robot‐assisted LDG and LLAR cases increased but at a rate lower than the prepandemic increase. Mortality and anastomotic leakage, two very important complications assessed in SMR, did not worsen during the pandemic compared to prepandemic levels.

**Conclusion:**

In Japan, laparoscopic surgery was safe and unaffected by the COVID‐19 pandemic, even in 2022, when the epidemic spread.

## Graphical abstract

Mortality and anastomotic leakage, two very important complications assessed in SMR, did not worsen during the pandemic compared to prepandemic levels. In Japan, laparoscopic surgery was safe and unaffected by the COVID‐19 pandemic even in 2022, when the epidemic spread.

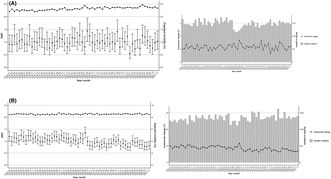

## 1 INTRODUCTION

Coronavirus disease 2019 (COVID‐19), first detected in December 2019 in Wuhan, China, is caused by severe acute respiratory syndrome coronavirus. During 2020, it quickly spread around the world.^1^ The COVID‐19 pandemic has significantly affected medical care throughout Japan, and its spread has continued to affect the medical situation, including the screening, diagnosis, and treatment of cancer.^2^, ^3^, ^4^ The Japan Surgical Society guidelines on COVID‐19 warned of the development of aerosols during laparoscopic surgery and that appropriate conditions, such as the use of a highly precise filter and effluent gas device, should be confirmed before it is performed.^5^ During the pandemic, insufficient numbers of medical materials and laparoscopic surgical instruments were available due to transportation difficulties. As shown by a Japan Society for Endoscopic Surgery survey, most surgical approaches to gastric and colorectal cancer are now by laparoscopy.

We previously reported the impact of COVID‐19 on laparoscopic surgery for gastric and rectal cancer.^5^, ^6^ The number of laparoscopic surgeries decreased in 2020 and remained low in 2021, without recovering, but the short‐term postoperative outcomes, such as the incidence of anastomotic leakage and mortality, remained unchanged from before the pandemic, proving that high‐quality surgery was being performed in Japan.

The shortage of medical supplies and the availability of surgical instruments during 2020 and 2021, as described above, began to gradually improve in 2022. Furthermore, 2022 was the third year of the pandemic, a time when vaccines were widely available and preoperative polymerase chain reaction (PCR) screening was widespread. It remains unclear whether the rates of complications were impacted by the COVID‐19 pandemic and whether mortality rates continued to be affected in 2022, which is considered the late phase of the COVID‐19 pandemic. Therefore, in the present study we aimed to clarify the impact of the COVID‐19 pandemic on mortality and morbidity in patients undergoing laparoscopic surgery for gastric cancer and rectal cancer in Japan in 2022 with comparison to the prepandemic period (2018) and pandemic period (2019–2021) by risk adjustment based on patient background characteristics.

## 2 METHODS

### 2.1 Patients

This study analyzed essential data extracted from the Japanese National Clinical Database (NCD), which is a nationwide registry system linked to the surgical board certification system since 2011. Information on data registration for the NCD system has already been reported.^7^, ^8^ By 2018, more than 5000 institutions had participated in this system, with annual registration of around 1.5 million surgical cases. All surgical cases are registered in the NCD, with morbidities and comorbidities, postoperative complications, and mortality also included.

As laparoscopic distal gastrectomy (LDG) and laparoscopic low anterior resection (LLAR) are the most common types of laparoscopic surgeries performed in Japan, these surgeries were selected for analysis.^6^, ^9^ Between 2018 and 2022, 122 450 LDG cases and 265 571 LLAR cases were registered in the NCD. This study excluded patients <18 y old and those undergoing emergent surgeries. In the LDG and LLAR groups, patients with benign disease, malignant disease of organs not including the stomach and rectum, diseases with unclear tumor depth (T) or node metastasis (N), and incomplete data for LDG and LLAR were excluded.

### 2.2 Classification of prefectures by degree of infection

The cumulative number of infected individuals per population at the end of 2022 was used to indicate the degree of infection in each prefecture in Japan. Based on the degree of infection, prefectures were classified into a high infection group or low infection group. The high infection group comprised the 12 prefectures of Aichi, Chiba, Fukuoka, Hokkaido, Hyogo, Kanagawa, Kyoto, Nara, Okinawa, Osaka, Saitama, and Tokyo, and the low infection group included all of the other prefectures.

### 2.3 Study endpoint

The primary outcome measure was identification of the affect that the COVID‐19 pandemic had on operative mortality and morbidity (anastomotic leakage) following LDG and LLAR. We defined operative mortality as a 30‐d mortality including postdischarge death or in‐hospital mortality during the index admission.

### 2.4 Clinical factors

The clinical factors assessed included age at surgery (<65, 65–75, and >75 y), sex (male or female), body mass index (BMI; ≤25 and ≥25 kg/m^2^), smoking history (Brinkman index: 0, <400, and ≥400), preoperative chemotherapy use, diabetes mellitus, habitual alcohol use, ischemic heart disease, congestive heart disease, hypertension, previous cerebrovascular disease, chronic obstructive pulmonary disease, requirement of preoperative dialysis, chronic steroid use, weight loss, bleeding disorder, preoperative blood transfusion, American Society of Anesthesiologists physical status (ASA‐PS: 1, 2, and 3–5), and clinical T, N, and M (distant metastasis) stages. Representative information on T, N, and M was extracted according to the American Joint Committee on Cancer TNM classification 7th edition. A between‐group comparative analysis of duration of surgery, intraoperative blood loss, and need for transfusion was also conducted.

The standardized mortality/morbidity ratio (SMR), defined as the ratio of observed number of patients to expected number of patients who experience complications, was calculated to examine trends in risk‐adjusted outcomes. Monthly rates of expected morbidity and mortality were calculated with a risk calculator devised on the basis of NCD data as reported in a previous study. Models based on logistic regression analyses^10^, ^11^, ^12^, ^13^ were constructed for operative mortality and anastomotic leakage following LDG and LLAR that incorporated variables such as sex, age, presurgery activities of daily living, comorbidities, and abnormal hematological findings. A significant difference was determined between the observed number and expected number of patients when the 95% confidence interval did not contain 1. Longitudinal graphs were constructed with Stata/BE 17 for Mac (StataCorp, College Station, TX, USA), and R v. 4.1.2 (2021; R Foundation for Statistical Computing, Vienna, Austria) was used for all statistical analyses.

The Institutional Review Board of Oita University approved the study protocol (approval number: 2444) and granted consent to perform this study.

## 3 RESULTS

### 3.1 Annual procedure numbers (2018–2022)

**FIGURE 1:**
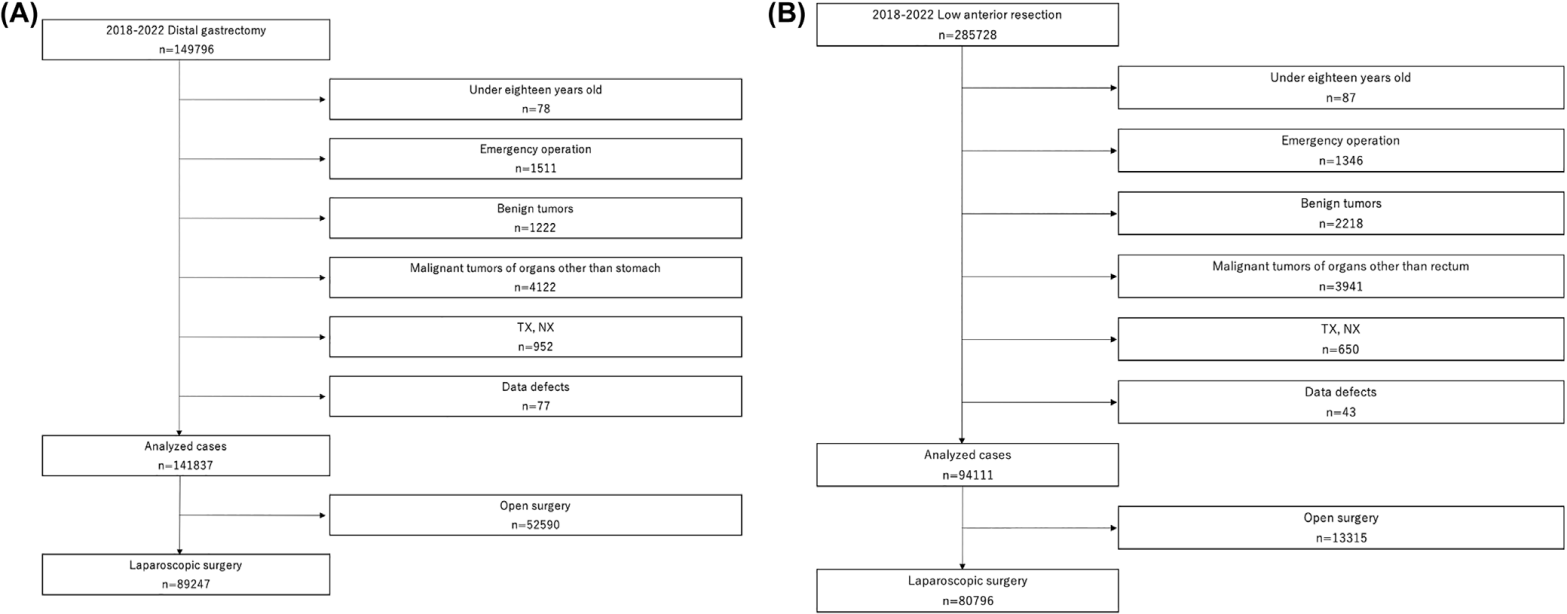
Flowchart of the patient selection process. (A) Laparoscopic distal gastrectomy. (B) Laparoscopic low anterior resection.

A flowchart illustrating the patient selection process during the analyzed study period of 2018 to 2022 is shown in Figure 1, with the numbers of procedures performed each year shown in Table 1. The numbers of cases treated by procedure in the high and low infection prefectural groups are listed in Table 2. In the high group, for DG, the number of open distal gastrectomies (ODGs) in 2022 was 82.4% of that in 2021 and 74.7% of that in 2020. The number of LDGs in 2022 was 98.7% of that in 2021 and 101.5% of that in 2020. The number of open low anterior resections (OLARs) in 2022 was 83.8% of that in 2021 and 67.8% of that in 2020. The number of LLARs in 2022 was 92.8% of that in 2019 and 90.2% of that in 2020. The number of robot‐assisted low anterior resections (RLARs) in 2022 was 124.5% of that in 2020 and 159.1% of that in 2020. In the low infection group, for DG, the number of ODGs in 2022 was 86.4% and 80.4% of that in 2021 and 2020, respectively. The number of LDGs in 2022 was 97.4% of that in 2021 and 95.5% of that in 2020. The number of OLARs in 2022 was 81.4% of that in 2021 and 70.0% of that in 2020. The number of LLARs in 2022 was 92.0% of that in 2021 and 90.9% of that in 2020. The number of RLARs in 2022 was 123.0% of that in 2021 and 165.7% of that in 2020.

**TABLE 1:** Numbers of operations performed.

	Number of operations (2018)	Number of operations (2019)	Number of operations (2020)	Number of operations (2021)	Number of operations (2022)	vs. 2018	vs. 2019	vs. 2020	vs. 2021
Distal gastrectomy									
Open	13 531	12 085	10 012	9199	7764	57.40%	64.20%	77.50%	84.40%
Laparoscopic	16 895	16 610	14 271	14 367	14 101	83.50%	84.90%	98.80%	98.10%
Robot‐assisted	1159	2102	2553	3213	3984	343.70%	189.50%	156.10%	124.00%
Total	31 585	30 797	26 836	26 779	25 849	81.84%	83.93%	96.32%	96.53%
Low anterior resection									
Open	3732	3159	2544	2126	1754	57.00%	67.30%	83.60%	82.50%
Laparoscopic	14 308	13 977	12 316	12 077	11 142	84.40%	86.40%	98.10%	92.30%
Robot‐assisted	1012	2448	3460	4503	5583	445.00%	183.90%	130.10%	124.00%
Total	19 052	19 584	18 320	18 706	18 479	96.99%	94.36%	100.87%	98.79%

**TABLE 2:** Numbers of operations performed according to degree of infection.

	Number of operations (2018)	Number of operations (2019)	Number of operations (2020)	Number of operations (2021)	Number of operations (2022)	vs. 2018	vs. 2019	vs. 2020	vs. 2021
High infection group									
Distal gastrectomy									
Open	6928	6176	5055	4586	3777	54.5%	61.2%	74.7%	82.4%
Laparoscopic	9844	9585	7931	8152	8046	81.7%	83.9%	101.5%	98.7%
Robot‐assisted	708	1306	1609	1976	2400	339.0%	183.8%	149.2%	121.5%
Low anterior resection									
Open	1905	1546	1227	993	832	43.7%	53.8%	67.8%	83.8%
Laparoscopic	8599	8450	7254	7046	6542	76.1%	77.4%	90.2%	92.8%
Robot‐assisted	640	1612	2283	2917	3633	567.7%	225.4%	159.1%	124.5%
Low infection group									
Distal gastrectomy									
Open	6603	5909	4957	4613	3987	60.4%	67.5%	80.4%	86.4%
Laparoscopic	7051	7052	6340	6215	6055	85.9%	85.9%	95.5%	97.4%
Robot‐assisted	451	796	944	1237	1584	351.2%	199.0%	167.8%	128.1%
Low anterior resection									
Open	1827	1613	1317	1133	922	50.5%	57.2%	70.0%	81.4%
Laparoscopic	5709	5527	5062	5001	4600	80.6%	83.2%	90.9%	92.0%
Robot‐assisted	372	836	1177	1586	1950	524.2%	233.3%	165.7%	123.0%

### 3.2 Trends in annual proportion of clinicopathological features (2018–2022)

**FIGURE 2:**
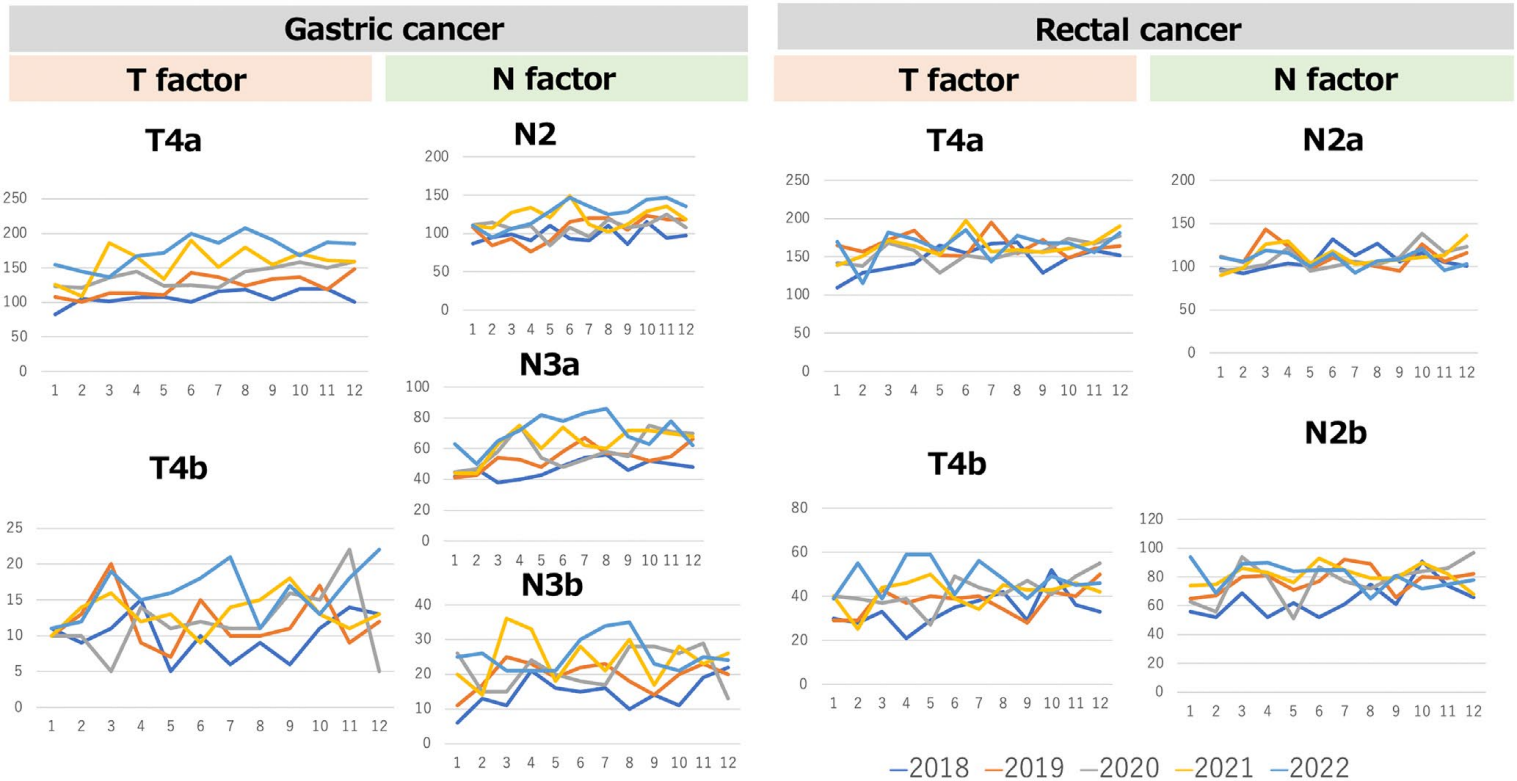
Monthly trends in tumor characteristics for each cancer type during the study period.

The monthly trends in tumor characteristics for gastric and rectal cancers are shown in Figure 2. We extracted data for July 2022, the month during which the seventh wave of COVID‐19 occurred and for which the daily number of domestic infections was highest from the beginning of the pandemic to the present.^14^ Trends in July across the 5 y of the pandemic revealed the following findings. Among patients receiving LDG, the percentages of T4b patients were 0.4% in 2018, 0.6% in 2019, 0.9% in 2020, 1.1% in 2021, and 1.4% in 2022; those of N2 patients were 6.0% in 2018, 7.1% in 2019, 8.2% in 2020, 8.4% in 2021, and 8.9% in 2022; and those of N3b patients were 1.1% in 2018, 1.4% in 2019, 1.5% in 2020, 1.6% in 2021, and 2.2% in 2022. Among the patients receiving LLAR, the percentage of T4b patients was 3.0% in 2018, 2.8% in 2019, 3.5% in 2019, 2.6% in 2021, and 4.2% in 2022; and that of N2b patients who received LLAR in July was 4.8% in 2018, 6.4% in 2019, 6.1% in 2020, 6.5% in 2021, and 6.4% in 2022.

### 3.3 Trends in standardized monthly ratios of mortality (2018–2022)

**FIGURE 3:**
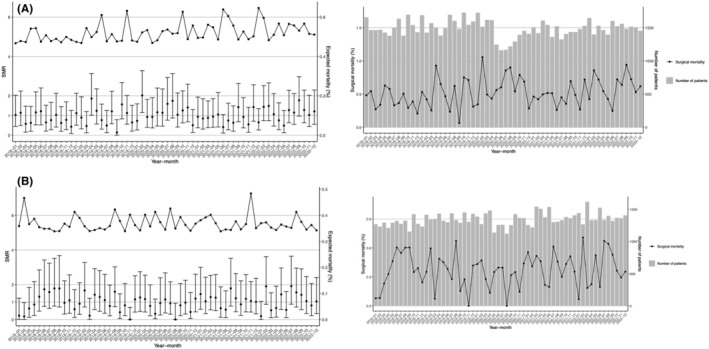
Mortality (standardized mortality ratio [SMR]). The SMR during each month of the study period is shown for (A) laparoscopic distal gastrectomy (LDG) and (B) laparoscopic low anterior resection (LLAR). Vertical lines indicate the SMR with the 95% confidence interval. Solid black line indicates the expected mortality rate.

The standardized mortality ratios for LDG and LLAR are shown in Figure 3A,B, respectively. The expected mortality ratio for LDG was about 0.5, and that for LLAR also remained at about 0.35. Both values were as low in the COVID‐19 infection period from 2019 to 2022 as in the prepandemic period.

### 3.4 Trends in standardized monthly ratios of anastomotic leakage (2018–2022)

**FIGURE 4:**
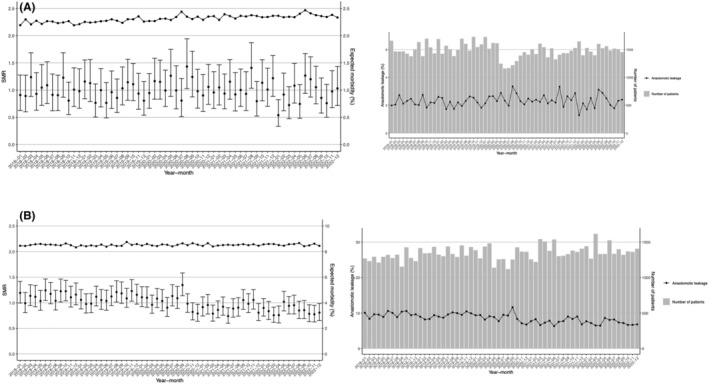
Morbidity for anastomotic leakage (standardized morbidity ratio [SMR]). The SMR for anastomotic leakage during each month of the study period is shown for (A) laparoscopic distal gastrectomy (LDG) and (B) laparoscopic low anterior resection (LLAR). Vertical lines indicate the SMR with the 95% confidence interval. Solid black line indicates the expected morbidity rate.

We next examined the incidence of the important complication of postoperative anastomotic leakage. During the study period, the mean actual incidence rates of anastomotic leakage were 2.2% for LDG and 8.5% for LLAR. After adjusting for various patient risks, we then calculated expected incidence rates to obtain the associated SMRs. Figure 4A,B show the trends of the expected morbidity rate and SMR for anastomotic leakage in the patients who underwent LDG and LLAR, respectively. For LDG, the incidence of anastomotic leakage remained at the same level as before the pandemic and showed no change during the study period, whereas LLAR showed a downward trend in 2022 compared with that before the pandemic.

## 4 DISCUSSION

As shown in this study, the numbers of LDG and LLAR decreased in 2020, the first year of the pandemic, and continued its decline in 2022 to the level in 2021. Neither a further decline nor a recovery trend in the number of cases was noted. However, robot‐assisted LDG and LLAR cases increased. Furthermore, the SMR and expected morbidity and mortality rates did not change significantly throughout most of the study period.

Compared to the early years of the spread of COVID‐19 infection in 2020–2021, the characteristics of 2022 are as follows: (1) the sixth and seventh waves occurred, resulting in an explosive increase in the number of infected patients; (2) vaccination began at the end of 2021; (3) severe cases of infection were a problem in 2021, but the number of severe cases decreased in 2022; and (4) preoperative PCR screening for COVID‐19 was thoroughly conducted at most facilities in Japan. These four characteristics are considered to be one of the important factors in analyzing the impact of COVID‐19 on medical care. The fact that robotic surgery for colon cancer in the colorectal cancer area was covered by insurance in Japan in 2022 must also be considered when evaluating the impact of COVID‐19 on medical care and the number of surgeries. While the spread of infection was explosive, the number of surgeries for gastric and colorectal cancer did not recover from 2021, but likewise did not decline further. In terms of surgical outcomes, the fact that mortality and the incidence of suture failure in both surgical procedures remained as good as the results before COVID‐19 infection suggests that the guidelines based on medical policy and those from academic societies in Japan were effective.

There was no difference in this above‐mentioned trend between endemic and nonendemic areas. The postoperative results even for surgery for more advanced cancers performed before and after the COVID‐19 epidemic remained favorable. Esophageal cancer showed no decrease in the number of operations before and after the COVID‐19 epidemic,^7^ nor were postoperative results for minimally invasive esophagectomy reported to worsen.^15^ For gastric and colorectal cancers, the numbers of operations decreased, but the numbers of advanced cancers increased as a result of a declining screening rate and reluctance to visit hospitals. As is inferred by the good short‐term results, laparoscopic surgery for gastrointestinal cancers can provide high‐quality outcomes, regardless of the spread of COVID‐19 infection.

From analysis of the data of 2022, it can also be inferred that vaccination, preoperative PCR screening for COVID‐19, and appropriate adherence to medical society and medical institution guidelines were effective. Even if COVID‐19 respreads and new viral infections occur in the future, minimally invasive surgery, as typified by laparoscopic surgery in Japan, is considered to be an effective technique for treating gastrointestinal tract cancer. However, a detailed study is needed to determine how surgical outcomes specific to robotic surgery, which is expected to become even more widespread in the future, and the process of introducing robotic surgery will affect the spread of COVID‐19 infection.

The present study is associated with several limitations. First, the number of surgical cases in 2022 was likely to be affected by the COVID‐19 infection pandemic. However, the number of gastric cancer surgeries has been declining in Japan, regardless of the spread of COVID‐19 infection, which is another important factor in the decline in the number of surgeries in addition to the spread of COVID‐19 infection. Therefore, there is a limitation in evaluating the impact of the COVID‐19 pandemic in terms of the number of surgeries, and future studies should take this into consideration. Second, the favorable SMR for anastomotic leakage may have been due to the effect of stoma creation. However, there are no data that have been validated by NCD items. Third, patients in whom surgery was avoided cannot be evaluated by NCD items. It is possible that cases of patients at high risk for death or complications were avoided, and thus showed stable outcomes. Finally, long‐term outcomes were not investigated. We consider that prognostic effects of a delay of surgical treatment due to triage will appear after several years. Thus, further studies will be needed to precisely evaluate the impact of the COVID‐19 pandemic on the outcomes of surgery for gastric and rectal cancer.

## 5 CONCLUSION

In conclusion, laparoscopic surgery was performed safely for gastric and rectal cancer in Japan and was not affected by the COVID‐19 pandemic. More evidence, such as long‐term outcomes, is needed to understand whom laparoscopic surgery can be performed on safely during future pandemics. In 2023, the government reclassified COVID‐19 infection from a Category 2 infectious disease (a notifiable novel infectious disease equivalent to tuberculosis and closely controlled by the government) to a Category 5 disease (a much less controlled non‐notifiable infectious disease equivalent to seasonal flu). However, the infection will not be completely eliminated, and it is expected that COVID‐19 will continue to persist even after its reclassification. Its impact on surgical treatment may be further complicated, and COVID‐19 infections will need to be carefully monitored and evaluated to determine any future impacts.

## AUTHOR CONTRIBUTIONS

**Masafumi Inomata:** Conceptualization; data curation; funding acquisition; methodology; project administration; supervision; writing – original draft. **Hideki Endo:** Conceptualization; formal analysis; methodology; validation. **Tomonori Akagi:** Conceptualization; data curation; investigation; project administration; writing – review and editing. **Hidefumi Shiroshita:** Conceptualization; methodology; project administration; writing – review and editing. **Shigeki Yamaguchi:** Conceptualization; project administration; writing – review and editing. **Susumu Eguchi:** Conceptualization; project administration; writing – review and editing. **Norihito Wada:** Conceptualization; project administration; writing – review and editing. **Yukinori Kurokawa:** Conceptualization; project administration; writing – review and editing. **Yosuke Seki:** Conceptualization; project administration; writing – review and editing. **Yoshiharu Sakai:** Conceptualization; project administration; writing – review and editing. **Hiroyuki Yamamoto:** Data curation; formal analysis; methodology; project administration; validation; writing – review and editing. **Yoshihiro Kakeji:** Conceptualization; project administration; writing – review and editing. **Yuko Kitagawa:** Conceptualization; project administration; supervision; writing – review and editing. **Akinobu Taketomi:** Conceptualization; project administration; supervision; writing – review and editing. **Masaki Mori:** Conceptualization; project administration; supervision; writing – review and editing.

## CONFLICT OF INTEREST STATEMENT

Hiroyuki Yamamoto and Hideki Endo are affiliated with the Department of Healthcare Quality Assessment at the University of Tokyo. The department is a social collaboration department supported by grants from the National Clinical Database, Intuitive Surgical Sarl, Johnson & Johnson K.K., and Nipro Co. Masafumi Inomata is an Editorial Board Member of the *Annals of Gastroenterological Surgery* dealing with the lower digestive tract. Susumu Eguchi is an Editorial Board Member of the *Annals of Gastroenterological Surgery* dealing with the hepato‐biliary‐pancreatic system. Yukinori Kurokawa is an Associate Editor of the *Annals of Gastroenterological Surgery* dealing with the upper digestive tract and has received lecture fees from Johnson & Johnson, Covidien Japan, Stryker, and MC Medical outside of the submitted work. Yoshihiko Kakeji is an Associate Editor of the *Annals of Gastroenterological Surgery* dealing with the lower digestive tract. Yuko Kitagawa is the Editor‐in‐Chief of the *Annals of Gastroenterological Surgery*. Masaki Mori is an Emeritus Editor‐in‐Chief of the *Annals of Gastroenterological Surgery*. The remaining authors declare no conflicts of interest for this article.

## ETHICS STATEMENT

Approval of the research protocol by an Institutional Reviewer Board: The study protocol was approved by the Institutional Review Board of Oita University (approval number: 2444).

Informed Consent: N/A.

Registry and the Registration No. of the study/trial: N/A.

Animal Studies: N/A.
